# Working from home and productivity under the COVID-19 pandemic: Using survey data of four manufacturing firms

**DOI:** 10.1371/journal.pone.0261761

**Published:** 2021-12-23

**Authors:** Ritsu Kitagawa, Sachiko Kuroda, Hiroko Okudaira, Hideo Owan

**Affiliations:** 1 Graduate School of Economics, Waseda University, Tokyo, Japan; 2 Faculty of Education and Integrated Arts and Sciences, Waseda University; The Research Institute of Economy, Trade and Industry, Tokyo, Japan; 3 Business School, Doshisha University, Kyoto, Japan; 4 Faculty of Political Science and Economics, Waseda University; The Research Institute of Economy, Trade and Industry, Tokyo, Japan; Universitat de Valencia, SPAIN

## Abstract

The coronavirus disease 2019 (COVID-19) pandemic has impacted the world economy in various ways. In particular, the drastic shift to telework has dramatically changed how people work. Whether the new style of working from home (WFH) will remain in our society highly depends on its effects on workers’ productivity. However, to the best of our knowledge, the effects of WFH on productivity are still unclear. By leveraging unique surveys conducted at four manufacturing firms in Japan, we assess within-company productivity differences between those who work from home and those who do not, along with identifying possible factors of productivity changes due to WFH. Our main findings are as follows. First, after ruling out the time-invariant component of individual productivity and separate trends specific to employee attributes, we find that workers who worked from home experienced productivity declines more than those who did not. Second, our analysis shows that poor WFH setups and communication difficulties are the major reasons for productivity losses. Third, we find that the mental health of workers who work from home is better than that of workers who are unable to work from home. Our result suggests that if appropriate investments in upgrading WFH setups and facilitating communication can be made, WFH may improve productivity by improving employees’ health and well-being.

## 1. Introduction

The coronavirus disease 2019 (COVID-19) pandemic has impacted the world economy in various ways. As one of the major changes, teleworking or working from home (WFH) has become widespread across countries. For example, Brynjolfsson et al. [1] suggest that in May 2020, approximately half of the workforce in the U.S. was WFH. Eurofound [2] showed that in July 2020, nearly half of all employees in EU countries worked from home. For Japan, the Cabinet Office [3] reported that the WFH percentage was 34.5% at the end of May 2020 (see also Morikawa [4] and Okubo [5]). Regarding other countries, see also Felstead and Rueschke [6], Pouliakas [7] and Delaporte and Pena [8]. While the WFH percentages may have varied across countries, two common features are observed: (1) many people reported that during the crisis, it was their first time WFH (for example, see [2, 5, 6]), and (2) the majority of workers WFH wished to continue the new working style even if there were no COVID-19 restrictions ([2, 3, 6]). This new global experience suggests that WFH may increase the welfare of workers and that the experience of WFH during the crisis may lead to growth in teleworking even after the crisis abates ([2]). However, the current evidence seems still mixed since several studies have found worsened psychological well-being associated with WFH (see [6] and Xiao et al. [9]), while enhanced well-being is reported in particular for those who are able to pursue their job at home (see Kroll and Nuesch [10], Bellmann and Hubler [11, 12]).

This pandemic-driven WFH has dramatically changed people’s way of work, and it is crucial to sustain production during this ongoing crisis. Whether the new style will remain in our society highly depends on its effects on workers’ productivity. However, the effects of WFH on productivity are still unclear (OECD [13]). For example, Bloom et al. [14] found that WFH had a positive effect on call center workers’ productivity and reduced turnover. While the paper ([14]) reported evidence based on data collected before the COVID-19 pandemic, Emanuel and Harrington [15] also found a positive effect on the productivity of call center workers during the COVID-19 crisis. On the other hand, Morikawa [4] showed that the mean WFH productivity relative to working at the usual workplace was approximately 60% to 70% in Japan, and 82% of workers reported a decline in productivity in a WFH environment during the COVID-19 crisis (Felstead and Rueschke [6] reported mixed results by analyzing broader occupations in the U.K.: see also [16–18]).

Several studies have also reported both positive and negative effects of WFH on productivity, depending on skills, education, tasks or industry. For example, Etheridge et al. [19] reported that in the U.K., women and those in low-paying jobs suffered the worst average declines in productivity (see also [20–22]). The paper also reported that declines in productivity are strongly associated with declines in mental well-being (see also Bartik et al. [23], Dutcher [24], De Sio et al. [25], Escudero-Castillo et al. [26] and Oakman et al. [27]). On the other hand, papers report positive characteristics of teleworking, such as ncreased efficiency and a lower risk of burnout (see, for example, Baert et al. [28]).

Physical health is also the mediating factor for the productivity effect of WFH, and musculoskeletal symptoms are often discussed as a problem with WFH. While Moretti et al. [29] and Yoshimoto et al. [30] document that workers suffer from musculoskeletal issues due to WFH during the pandemic (see also [31]), Aegerter et al. [32] find the effect of WFH on neck pain and disability to be limited, and Seva, Tejero, and Fadrilan-Camacho [33] show that musculoskeletal symptoms had no significant effect on the productivity of telecommuters. In summary, although there has been a rapid accumulation of studies on WFH and productivity, the reported evidence is mixed, and we believe that additional evidence on when WFH is productivity-enhancing is needed.

In this study, we try to contribute additional evidence on the effects of WFH by using data from our original employee-level survey conducted in cooperation with four large listed manufacturing companies in Japan from April to June 2020. Specifically, we assess within-company productivity differences between those who work from home and those who do not, along with identifying possible factors of productivity changes due to WFH.

On April 7, 2020, the Japanese government declared a countrywide state of emergency. Although the state of emergency ended on May 25, the request for self-restraint on movement between prefectures was extended until June 19. In the meantime, the government asked firms to let workers work from home as much as possible. According to the panel survey conducted by the Japan Institute for Labour Policy and Training (JILPT) (2020), the number of WFH workers rapidly increased from early April and peaked in the second week of May 2020. It then started to decline after the state of emergency was lifted at the end of May and dropped significantly by the end of July. Notably, although the government declared a state of emergency, it was only *on a request basis* and was not mandatory; therefore, the final decision on whether to introduce WFH was made completely at the discretion of employers. Moreover, many Japanese firms allowed each workplace to individually decide whether to use WFH. Therefore, even in the same firm, while workers in some units worked entirely from home, workers in other units had to commute to the office even though both groups of workers performed similar tasks. The variations in WFH within the same company enable us to investigate whether there are productivity losses or gains due to WFH. However, because companies and middle managers had the discretion to comply with or to defy the official request, the decision to opt for WFH may be endogenous if workers with specific unobserved traits or roles in the workplace tended to be chosen for WFH. We mitigate this concern over endogeneity in two ways, which we explain as part of the empirical strategy in Section 3.

The survey we use includes questions on subjective productivity and the perceived factors of productivity losses, allowing us to investigate the possible determinants of deteriorations in productivity. It also contains questions on mental health and the perceived advantages and disadvantages of WFH, making it possible to examine the relationship between WFH and workers’ mental health.

Our major contributions are threefold. First, using employee survey data with relatively high response rates, we exploit the heterogeneities among workers within the same companies. Specifically, we identify the effects of WFH on productivity within the same company and within the same occupation, which vary depending on the number of days spent WFH. Focusing on specific companies also allows us to exclude the effects of differences in productivity, labor-management relationships, and organizational support for WFH among firms. Based on our analysis, workers who worked from home experienced a productivity decline compared with those who did not. Second, owing to the rich information available in our original surveys, we could identify the potential factors that determine deteriorations in productivity due to WFH. We find that poor WFH setups and communication difficulties are the major reasons for productivity losses. In addition, although the reasons above are common features of all occupations, we find that the major reasons that reduce productivity the most differ by occupation. Third, we complement our findings by analyzing the impact of WFH on mental health. We find that the mental health of WFH workers is significantly better than that of workers who are unable to work from home. Our results suggest that if appropriate investments in upgrading WFH setups and facilitating communication can be made, WFH may improve productivity by improving employees’ health and well-being.

One caveat is that our sample is not representative of Japanese workers, and this should be noted as a limitation, especially when discussing policy implications. Our sample is limited to those in the manufacturing sector. The average worker in our sample is more educated, working in larger and more male-dominated organizations, and more likely to be in technical and professional jobs than the general population. However, the sample includes many occupations from those with the highest to the lowest likelihood of working from home, which makes it suitable for examining the heterogeneity of the effect of working from home in the same management and business environment.

The remainder of this paper is organized as follows. Section 2 describes our data, and Section 3 presents our quantitative methods. Section 4 explains the results, and Section 5 concludes.

## 2. Data

We use data retrieved from our original survey on WFH productivity during the COVID-19 pandemic, which was conducted in cooperation with four listed manufacturing companies in Japan (Companies A, B, C, and D) from April to June 2020. Companies A, B, and D are chemical manufacturing companies, while Company C is an automobile manufacturing company. Companies A, B, and D have approximately 8,000, 7,000, and 27,000 employees, respectively, while Company C has more than 30,000 employees on a consolidated basis. The survey was conducted after each company responded to the authors’ proposal to examine the effect of working from home using a common questionnaire. Therefore, each company’s survey period, target, and questionnaire are somewhat different from the others because they were initiated by each company’s management and tailored to its needs. Some questions were modified to be consistent with similar questions included in its regular employee survey (see Table 1 for major differences across companies). The employees were told that the responses collected would be analyzed anonymously by the department in charge and that only aggregated figures for each organization would be shared with their superiors following each company’s data protection and privacy policy. The authors were given access to the anonymized dataset after the companies’ internal use. This research has been judged as not requiring review by the institutional review board of Waseda University, where the corresponding author works.

**Table 1:** Comparison of employee surveys for companies A-D.

	Company A	Company B	Company C	Company D
Sample	All employees	All employees (incl. subsidiaries)	All eployees (excl. blue-collar workers)	All employees (incl. subsidiaries)
Reference Period	From April 1 to the date of response May 20–26	From May 11 to the date of response May 20-June 3	From May 11 to the date of response June 17–26	From April 1 to the date of response April 23-May 7
Survey Period				
Response Rate	91%	43%*	72%	43%
Pre-COVID presenteesm	Measured retrospectively	Measured retrospectively	Measured retrospectively	Measured in February 2020
Missing information	Mental health state, WFH days before April, perceived advantages of WFH			
Different questions	No temporal range is specified for the pre-COVID presenteesm.			Different scale of presenteeism/modified list of perceived causes of lower productivity and perceived advantages of WFH
Days spend WFH per week(%)				
5 days	8.1	22.5	18.4	21.2
3-4days	14.9	9.9	31.4	17.0
1-2days	25.0	19.6	41.0	18.7
None	52.0	48.0	9.2	43.1
Occupational Compositions (%)				
Corporate function	37.9	27.1	15.1	26.0
Sales	22.0	11.6	13.2	26.5
R&D	18.6	24.6	40.8	16.6
Production	21.4	36.6	31.0	9.9
Number of observations	2877	3458	3989	12941
% of those who worked from home in early March	N.A.	35.2	20.1	10.7

Note

(*): the reponse rate for Company B is calculated based on the information for the parent company. It is unknown how many employees were targted for the survey among subsidiaries.

The survey was administered to both white- and blue-collar employees (Companies A, B, and D) or white-collar employees (Company C). Hence, the Company C sample does not include blue-collar workers, who regularly worked at the factory during the survey period, resulting in the smaller proportion of “no WFH” responses compared to the other companies. The employees of Companies B and D also included those of subsidiary companies. All employees of the four companies were asked to complete the survey. The survey included questions on topics such as the number of days spent WFH per week, productivity (presenteeism; details will be explained in Section 2.1.1.) before and after the state of emergency, the perceived causes of productivity losses, the respondents’ mental health status (details will be explained in Section 2.1.2.), the perceived advantages and disadvantages of WFH, and the respondents’ occupation, job grade, division, and basic individual characteristics. The response rates vary across the companies, ranging from 43% to 91%. The total sample size was 24,175, which fell to 22,815 after excluding invalid responses. Because the survey asked about the respondents’ productivity level both before and after the state of emergency, our analyses could rule out the time-invariant component of individual productivity.

The survey included a question on the number of days spent WFH per week during the reference period. We consolidated the answers into four categories based on the number of days spent WFH: none, once or twice, three or four times, and five times a week (i.e., exclusively WFH). Table 1 shows the percentage of employees who worked from home by the number of days worked from home per week on a company-by-company basis. It shows that among employees within the same company, there is variation in the number of days spent WFH.

Moreover, the percentage of workers who completely worked from home, i.e., those who worked from home five days a week, ranged from approximately 8% to 22% across the four companies. On the other hand, the figures show that approximately 40% to 50% of employees of Companies A, B, and D and 10% of employees of Company C worked entirely at the office. Note that this share of employees, not WFH, is low for Company C because it asked only white-collar employees to complete the survey.

### 2.1 Outcome variables

#### 2.1.1. Productivity

In our survey, productivity was measured based on answers to the modified version of the Health and Work Performance Questionnaire (HPQ), which was developed by the World Health Organization (WHO) and used to measure subjective productivity (presenteeism). Our productivity measurement was conducted based on two-stage questions following the WHO-HPQ. The first item asked respondents the following retrospective question: “(*o*)*n a scale from 0 to 10 where 0 is the worst job performance anyone could have at your job*, *5 is the performance of average workers*, *and 10 is the performance of a top worker*, *how would you rate your usual job performance (in the one-year period) before the declaration of the state of emergency*?” This item aimed to determine the average level of productivity of individual employees in the pre-COVID-19 period. In the questionnaire used for Company A, however, the phrase “in the one-year period” in the parentheses was not included. This means that pre-COVID presenteeism may be underestimated for Company A if a much shorter preperiod is considered by its employees.

The second question asked respondents to also apply a “0 to 10” scale to grade their overall job performance for a specific period during the pandemic (the actual period varies from company to company between April 2020 and June 2020). Taking the difference between the answers to these two questions, we calculated the changes in productivity before and after the state of emergency, which allows us to account for unobserved heterogeneity among workers. Regarding Company D, the simplified University of Tokyo version of the one-item presenteeism scale (Presenteeism-UT), which aimed to reduce the number of questions based on the HPQ, was used. For Company D, the employee survey was conducted twice, first in early March 2020 before the state of emergency was declared and again in April 2020. Therefore, unlike the other companies for which presenteeism before the state of emergency was evaluated in a retrospective manner, for Company D, presenteeism was measured at two time points—before and after the state of emergency. Specifically, the Presenteeism-UT asked employees to “Suppose that 100% is your work performance when you are neither sick nor injured. Please evaluate your current work performance.” For the April survey, the question was changed to “Suppose that 100% is your work performance when you are neither sick nor injured before the state of emergency. Please evaluate your current work performance after April 8.” We standardized the responses to a 0–10 scale by dividing by 10.

We understand that the retrospective method used at Companies A-C might be problematic when the measurement error for the preoutcome correlates with the independent variable of interest, the number of days spent WFH per week (hereafter WFH in short), thus biasing the estimates for WFH coefficients. WFH may correlate positively or negatively with productivity changes. For example, those who worked from home may tend to understate their past productivity level, thereby overstating the productivity growth after the pandemic to pretend that they worked hard even in the pandemic. In this case, the coefficient estimate for WFH is overestimated in the presence of bias in the dependent variable. Alternatively, workers may overstate their past productivity level and understate productivity growth after the pandemic so that they can accuse their productivity decline to the pandemic. If this situation is more serious for those who worked from home, the coefficient estimate for WFH would be downward biased. Although we do not know which type of retrospective error is potentially more likely to arise, we believe that the possibility of such biases is relatively limited since in all four firms, the employees were explained that the individual responses were anonymized and would not be disclosed to the superiors. Therefore, workers should have had very limited incentive to manipulate their productivity level, if any.

We use this presenteeism measure as one of our main outcome variables. Higher values indicate less presenteeism (i.e., higher productivity).

#### 2.1.2. Mental health index

Another main outcome variable of this paper is employees’ mental health. In the survey, we asked respondents to “(p)lease answer the following questions concerning your health since [the start date of the reference period]” along with the following three questions about workers’ mental health: “I have been depressed,” “I have felt weary or listless,” and “I have felt worried or insecure.” The respondents were asked to choose from four options: “almost always,” “often,” “sometimes,” and “almost never.”

In 2015, the Japanese Industrial Safety and Health Law was amended to mandate firms with 50 or more employees in the workplace to conduct a Stress Check Program once a year to screen high psychosocial stress. The Brief Job Stress Questionnaire (hereafter, the BJSQ) is highly recommended to firms by the Ministry of Health, Labour and Welfare for screening. Using data of 7356 male and 7362 female employees in a financial service company who completed the BJSQ, Tsutsumi et al. [34] report predictive validity of the BJSQ by finding that employees identified as high stress using the BJSQ had significantly elevated risks for long-term sickness absence by the one-year follow-up. Note that the three questions used in this paper to measure employees’ mental health are the same as those included in the BJSQ. We coded these responses on a 1 to 4 scale and reduced the total scores from the three questions into one dimension by using correspondence analysis with the dimension with the highest eigenvalue being the mental health index. Correspondence analysis reduces the dimension of scales among a set of qualitatively similar categorical variables (see, for instance, [35]). Higher values indicate better mental health. This index is highly correlated with the simple sum of the total Likert-based scales (the correlation coefficient is approximately 0.95 across firms). Note that this variable is not available for Company A.

### 2.2. Covariates of interest

#### 2.2.1. Perceived factors affecting productivity and mental health

The survey also asked respondents who worked from home during the reference period to choose potential factors that caused declines in their productivity. Specifically, the respondents were asked the following multiple-choice question: “what factors, if any, do you think lower productivity when working from home?” The choices were “*the inability to retrieve data from outside of the office because of security*,” *“the inability to use exclusive equipment that is available only at the office*,*” “poor WFH setups (e*.*g*., *do not have own office space)*,*” “lack of articulate orders and/or poor support from superiors*,*” “poor workplace communication*,*” “poor communication with clients*,*” “fatigue from an excessive workload*,*” “not feeling well physically (stiff shoulders*, *back pain*, *etc*.*)*,*” “feeling mentally under the weather*,*” and “having distractions or responsibilities to deal with (such as kids who want attention*, *nursing care for parents*, *and other family responsibilities)*.*”* Note that some of the choices were missing in the questionnaire for Company D.

In the survey, we also asked WFH employees additional multiple-choice questions about workers’ perceived advantages and disadvantages of WFH on mental health. Specifically, we asked, “*While working from home*, *did you find any advantages (disadvantages) of WFH that may have improve (worsen) your stress*, *if any*?” The choices of advantages were “*no distractions and a quiet environment that facilitates a greater focus on work*,” “*can avoid frequent and/or unnecessary conversations with coworkers*,” *“free from stress caused by annoying relationships with coworkers and bosses*,*”* “*improvement in IT skills*,” “*zero commuting and saving time on getting ready for work*,” “*being able to wear casual clothes*,” “*less fatigue and having a healthier condition*,” “*eating healthier meals*,” “*spending more time exercising*,” “*reducing alcohol consumption*,” “*having extra time for sleep and rest*,” “*less smoking*,” “*having extra time with family and friends*,” “*the ability to fit in household chores*, *parental care*, *and extra time with kids*,” “*better family relationships*,” and “*finding new hobbies due to the constraints on going out*.”

The choices of disadvantages were “*project delay*,”, “*lack of coordination/communication in workplace*”, “*poor IT environment*,” “*musculoskeletal pain*,” “*eye strain*,” “*migraine*,” “*having to prepare meals*,” “*eating unhealthier meals*,” “*spending less time exercise*,” “*weight gain*,” “*increasing alcohol consumption*,” “*snacking*,” “*more smoking*,” “*disturbed sleep*,” “*decreased conversation and feeling alone*,” “*childcare due to school closure*,” “*nursing care for parents*,” “*worse family relationships*,” “*constraints on going out*.” Some items in the advantage and disadvantage are conceptually paired in a sense that they represent the opposite of each other. In such a case, we take the difference between the advantage and disadvantage items and use it as an advantage variable. The variables created in this manner are “*can avoid unnecessary communication*,” “*free from annoying relationships with coworkers and bosses*,” “*exercising more*,” “*drinking less alcohol*,” “*smoking less*,” “*better sleep*,” “*better diet*,” “*better family relationship*,” and “*enjoying staying home*.”

#### 2.2.2. Functional roles

Using the occupational classification of each employee, we categorized the employees into four functional roles: *corporate*, *sales*, *R&D*, and *production*. Production included not only blue-collar employees who engage in the production process but also white-collar employees who manage production and quality control. In the following, we divide our observations into subsamples by these four categories to investigate whether the possible causes that reduce WFH productivity may differ across functional roles.

S1 Table presents the descriptive statistics of each company.

## 3. Empirical strategy

### 3.1. Main model

We are interested in identifying the impact of the individual’s WFH status on the outcome variable (*y*_*ijt*_) for individual *i* at division *j* in firm *k* at time *t*. We start with a simple linear model:

$$y_{ijkt}=z_{ijkt}\beta +X_{ijkt}\gamma +\epsilon_{ijkt}$$ (1)

where *z*_*ijkt*_ is the number of days spent WFH per week or a vector of dummies (*wfh2d*, *wfh4d*, *wfh5d*); *X*_*ijkt*_ is a vector of individual and division-specific characteristics; and *ϵ*_*ijkt*_ is an error term. *wfh2d*_*i*_, *wfh*4*d*_*i*_, and *wfh*5*d*_*i*_ indicate the number of days spent WFH per week, i.e., “once or twice,” “three or four times,” and “five times (exclusively),” respectively. The reference is none (zero WFH days). A vector of dummies (*wfh2d*, *wfh4d*, *wfh5d*) is used when we suspect a nonlinear relationship between the frequency of WFH and the outcome.

This study used different identification strategies for the presenteeism and mental health variables. For presenteeism, our survey asked for a subjective assessment of productivity in March (i.e., prior to the declaration of the state of emergency) and in April or May (i.e., after the declaration), and we had one observation point for mental health. We first explain our approach to the former in this section and to the latter in the next section.

We can identify *β* using ordinary least squares (OLS) if the WFH term is orthogonal to the error term, conditional on individual characteristics. This assumption is likely to be violated if workers with specific unobserved traits or roles in the workplace tend to be chosen for WFH. If companies are more likely to allow more productive workers to work from home, the estimated *β* will be overestimated. Likewise, if less productive workers volunteer to work from home disproportionately more often than more productive workers, then the estimate for *β* will be underestimated.

In our case, the shock to WFH adoption was mostly exogenous. Similar to the context of previous studies on the impact of WFH after the pandemic, the declaration of a state of emergency in Japan had a large and less expected impact on WFH adoption. According to Table 1, quite a large number of workers worked from home owing to the government’s request in April. More than half of the employees in our sample worked from home at least once a week. Importantly, however, the government’s WFH request was not mandatory. Because companies and middle managers had the discretion to comply with or to defy the official request, the decision to opt for WFH may still be endogenous.

We overcome this concern over endogeneity in our subjective productivity measure in two ways. First, we take the first difference in Eq (1) to rule out unobserved time-invariant individual and division-specific characteristics in the error term, which are correlated with factors affecting the WFH choice.

$$\Delta y_{ijkt}=\Delta z_{ijkt}\beta +\Delta X_{ijkt}\gamma +\Delta \epsilon_{ijkt}$$ (2)

where Δ is the first-difference operator.

As a result, our main sample is reduced to a cross-section of the first-differenced outcome variable. Δ*z*_*ijkt*_ is the difference in the number of days spent WFH during the period between the two surveys, which is denoted by *wfh_dif*. For Company A, information on the number of days spent WFH before April is lacking. We replace Δ*z*_*ijkt*_ with *z*_*ijkt*_ under the assumption that a very small number of employees worked from home for a limited number of days before April.

As shown below, most covariates in *X*_*ijkt*_ do not have much time series variation, which means that most values in Δ*X*_*ijkt*_ are zero. Additionally, although time-invariant individual and division-specific characteristics are ruled out by taking the first difference, they might still contribute to selection bias because they are likely to be correlated with time-varying unobservables that affect both the WFH choice and the outcome. For these reasons, we replace Δ*X*_*ijkt*_ with *X*_*ijkt*_ in Eq (2). Thus, our baseline model is as follows:

$$\Delta y_{ijkt}=\Delta z_{ijkt}\beta +X_{ijkt}\gamma +\Delta \epsilon_{ijkt}$$ (3)

In particular, we include the following terms as *X*_*ijkt*_: a female dummy, age category dummies, and dummies for job grades and divisions. Including dummies for job grades and divisions in Eq (2) essentially allows us to control for separate trends across different job levels and divisions. Controlling for such trends is important in the analysis of WFH after the pandemic because a worker’s occupation and functional and technical roles within the organization could correlate with her superior’s WFH choice for her. In other words, by including dummies for job grades and divisions, the coefficient *β* is identified mainly based on the variation within the division and job level where the variation in WFH is primarily caused by the preference and management style of the worker’s supervisor, which is less likely to be correlated with the worker’s productivity.

To the extent that our estimation model controls for the selection bias arising from such endogenous adoption of WFH, the estimate of *β* represents the causal impact of WFH adoption. One cause for concern is that some employees were transferred across divisions during the reference period. However, their functional roles rarely changed after the transfer, and the effect of the division within the same functional role was not expected to differ substantially.

Another issue that we encounter is that the measurement of presenteeism is not necessarily consistent with the measurement of WFH. In the default questionnaire that we used, presenteeism was assessed for a one-year period before the declaration of the state of emergency, while the frequency of WFH was assessed for a one-week period in early March. The measurement period for the two is consistent for the question asked for the postdeclaration period. To mitigate the bias due to this time inconsistency, we add *z*_*ijt*_ as a control in some specifications. That is, we estimate the following:

$$\Delta y_{ijkt}=\Delta z_{ijkt}\beta_{1}+z_{ijkt}\beta_{2}+X_{ijkt}\gamma +\Delta \epsilon_{ijkt}$$ (4)

The equation will be estimated company by company (companies A, B, C, and D) so that betas will be company-specific estimates of the productivity change associated with the WFH change.

### 3.2. Model for mental health

As discussed above, for our mental health variable, we have one observation point. Thus, taking the first difference is not feasible. There are two major confounding factors for the relationship between mental health and WFH. First, a worker’s low ability or productivity might make it difficult for his superior to allow him to work from home, and at the same time, his low evaluation could harm his mental health. Second, jobs requiring many face-to-face interactions or heavy responsibilities might not only prohibit WFH but also place a greater mental burden on workers.

To address such possible confounding factors, we include both pre-COVID productivity and division/job level dummies as controls. Controlling for differences in workers’ workplace and job level also allows us to account for technical or operational reasons underlying the WFH choice.

Furthermore, we argue that for mental health, endogeneity of WFH is less of a concern than for productivity. Namely, it is unlikely that workers with a specific mental health condition tend to be chosen for WFH because a person’s mental health condition is not known to her supervisor until it has deteriorated so much that her productivity has started being seriously affected or her doctor’s recommendation of sick leave or a job transfer is submitted. Even if the supervisor knows her subordinate’s mental health condition before it becomes this bad, it is not a priori obvious whether choosing WFH will be good or bad for her health.

With all efforts to reduce potential confounding factors, we estimate Eq (1) using OLS to make causal interpretations. However, we still cannot rule out the possibility of some bias due to selection (see, for example, Bubonya et al.[36]). It might be the case that employees whose mental status was most seriously damaged by WFH were less likely to respond to the survey request. In this regard, although we think the magnitude of potential selection bias should be smaller than other survey approaches taken in the literature, such as web-based online surveys, the results below shall be observed with reservations.

### 3.3. Analysis using the WFH sample

Some survey questions, such as the item asking about the perceived factors of productivity declines, were asked only to workers who worked from home during the reference period. Furthermore, the answer to the question is likely to be correlated with the frequency of WFH. Therefore, the OLS estimates of Eq (3) for presenteeism or Eq (1) for mental health are biased if

$$E[\Delta \epsilon_{ijkt}|X_{ijkt},\;\Delta z_{ijkt},\;d=1]\neq 0$$

or

$$E[\epsilon_{ijkt}|X_{ijkt},\;z_{ijkt},\;d=1]\neq 0,$$

respectively, where *d* denotes a dummy for WFH at least one day a week.

Given our previous discussion, we predict that the OLS estimates of the first-difference equation for presenteeism might be biased due to the endogeneity of WFH if unobservable factors that separate trends of presenteeism are correlated with the decision to work from home. To investigate our predictions, we have estimated both OLS and type II Tobit models (models with sample selection biases).

Note that we cannot take the same approach to sample selection due to worker decisions not to respond to survey requests because we do not have access to worker characteristics information for those who did not answer the questionnaire.

## 4. Results

### 4.1. Frequency of WFH and productivity

First, we estimate Eq (2) without control variables to observe how the frequency of WFH affects productivity. The results are shown in Table 2. Note that the variable *wfh_dif* is missing for Company A because information on the number of days spent WFH before April is lacking. The coefficient estimates of the difference in the number of days spent WFH for Companies B-D and the WFH dummies for Company A are all significantly negative.

**Table 2:** Regression of productivity changes on WFH.

	Company A	Company B	Company C	Company D
	*prsnt_dif*			
				
*wfh_5d*	-0.321***	-	-	-
	(0.104)	-	-	-
*wfh_4d*	-0.597***	-	-	-
	(0.0956)	-	-	-
*wfh_2d*	-0.400***	-	-	-
	(0.0653)	-	-	-
*wfh_dif*	-	-0.0811***	-0.0350***	-0.249***
	-	(0.0245)	(0.0100)	(0.0349)
Constant	-0.0304	0.0517	-0.711***	-0.413***
	(0.0380)	(0.0400)	(0.0472)	(0.141)
Divisions	No	No	No	No
Job grades	No	No	No	No
Observations	2,798	3,404	3,989	10,753
R-squared	0.037	0.005	0.003	0.044

Robust standard errors in parentheses.

*** p<0.01

** p<0.05

* p<0.1.

In summary, the results indicate that workers who worked from home experienced declines in productivity compared with those who did not. This adverse effect was considerably large for Company D, which may have resulted from the fact that the survey was conducted in late April, two weeks after the declaration of the state of emergency. At that time, many employees were forced to work from home without full preparation, which may have temporarily resulted in a large decline in productivity.

Table 3 shows the full model including other explanatory variables (i.e., Eq (4)). For Company B, the first difference of the WFH days becomes statistically insignificant. On the other hand, although the magnitude of the estimates decreases, the frequency of WFH still negatively affects productivity for Company D even after controlling for various individual and job characteristics. Note that the level of WFH dummies is negative for both Companies B and D. For Company C, the magnitude of the first difference becomes even larger. However, the WFH dummy of 5 days is positive and statistically significant. We will reconsider this in the subsample analysis below.

**Table 3:** Regression of productivity changes on WFH with controls.

	Company A	Company B	Company C	Company D
	*prsnt_dif*			
*wfh_5d*	-0.223	-0.376***	0.437**	-0.658***
* *	(0.133)	(0.126)	(0.174)	(0.212)
*wfh_4d*	-0.453***	-0.403**	0.107	-0.887***
* *	(0.110)	(0.158)	(0.144)	(0.169)
*wfh_2d*	-0.336***	-0.168	-0.0452	-0.792***
* *	(0.0877)	(0.126)	(0.0980)	(0.135)
*wfh_dif*		-0.00377	-0862***	-0936***
* *		(0.0298)	(0.0183)	(0.0194)
*female_wo_child*	0.0222	0.0272	0.130	0.281***
* *	(0.0702)	(0.0973)	(0.0870)	(0.0702)
*female_w_child*	-0.0677	-0.117	0.106	0.146
* *	(0.163)	(0.132)	(0.198)	(0.145)
*age30*	-0.235**	-0.118	-0.323***	-0.235***
* *	(0.0934)	(0.127)	(0.0894)	(0.0653)
*age40*	-0.244***	0.0841	-0.192**	-0.399***
* *	(0.0784)	(0.126)	(0.0946)	(0.0786)
*age50*	-0.226**	0.0540	-0.118	-0.415***
* *	(0.0834)	(0.110)	(0.110)	(0.0869)
*age60*	-0.277	-0.131	0.0311	-0.628***
* *	(0.176)	(0.137)	(0.107)	(0.165)
Constant	-0.0737	-0.0647	-0.554***	3.399***
	(0.149)	(0.150)	(0.123)	(0.192)
Observations	2,798	2,812	3,720	10,690
R-squared	0.065	0.038	0.067	0.154

Robust standard errors in parentheses.

*** p<0.01

** p<0.05

* p<0.1.

The controls include job grades and sections.

The estimated negative effects of WFH should be interpreted carefully because our dependent variable is measured subjectively and subject to measurement error due to retrospective bias. This is particularly worrisome if workers tend to overstate their past productivity level and thus understate productivity growth after the pandemic. However, we believe that the workers’ inventive to manipulate the report is limited due to anonymous treatment of the responses, as we discussed earlier. Furthermore, the consistent results across all four companies suggest that the specificity of workers’ incentives in given situations is unlikely to have affected our results.

The full model offers another causal parameter worth mentioning. The productivity losses are greater for employees in their 30s, 40s, and 50s in Companies A, C, and D. Young workers are not significantly affected by the shift to WFH presumably because (1) they are more familiar with online communication and recent information technology than their older counterparts and (2) they are assigned more specialized or solo tasks requiring less coordination; thus, their productivity is less constrained by WFH. These results may provide evidence that, on average, employees experienced declines in productivity from WFH. Below, we investigate what factors caused such declines in productivity.

### 4.2. Causes of productivity losses

To identify the causes underlying the productivity losses, we add as explanatory variables the responses to the question of what factors the respondents perceived as causing their productivity to decline. For Company D, slightly different wording was used for some questions, but what was being asked was essentially the same. However, a few questions were not available. Accordingly, “the inability to retrieve data” and “having responsibilities (childcare and/or nursing care)” are missing for Company D. Here, the sample is restricted to those who worked from home at least one day per week after the state of emergency. Any factors that are strongly correlated with productivity losses should be the main mechanism underlying the drop in productivity.

Table 4 reveals two important common channels. First, “*poor WFH setups*” have significantly negative coefficients for all companies, and “*the inability to retrieve data from outside the office*” is also negatively correlated with changes in productivity for Companies A and B. These results indicate that the lack of sufficient infrastructure for WFH hinders employee performance. Second, *“poor workplace communication” and “poor communication with clients”* are significantly negative for almost all companies. This result implies that new communication applications such as social networking services (SNSs), chat apps and conference calls cannot easily replace traditional communication methods such as face-to-face communication or phones and their role in meeting spontaneous, simultaneous or urgent needs for communication.

**Table 4:** Regression of productivity changes on the perceived factors of productivity losses.

	Company A	Company B	Company C	Company D
	*prsnt_dif*			
Inability to retrieve data	-0.459***	-0.341***	-0.0596	-
	(0.157)	(0.0694)	(0.0557)	-
Inability to use exclusive equipment	-0.589***	-0.0787	-0.168***	-
	(0.0975)	(0.116)	(0.0560)	-
Poor WFH setups	-0.536***	-0.506***	-0.415***	-0.641***
	(0.162)	(0.0585)	(0.0590)	(0.0767)
Lack of support and/or instruction from the supervisor	-0.144	-0.256	-0.0553	-
	(0.274)	(0.195)	(0.0660)	-
Poor workplace communication	-0.503***	-0.0906	-0.387***	-0.148**
	(0.136)	(0.0950)	(0.0504)	(0.0610)
Poor communication with clients	-1.028***	-0.382***	-0.114*	-0.517***
	(0.101)	(0.0964)	(0.0685)	(0.0961)
Fatigue from an excessive workload	-0.717	0.444***	0.0449	-
	(0.604)	(0.140)	(0.0992)	-
Not feeling well physically	-0.111	0.174*	-0.0480	0.334***
	(0.241)	(0.0965)	(0.0682)	(0.0530)
Feeling mentally under the weather	-0.306	-0.372***	-0.0949	0.276***
	(0.316)	(0.109)	(0.0937)	(0.102)
Having responsiblities (childcare and/or nursing care)	-0.985***	0.414	-0.284***	-
	(0.335)	(0.324)	(0.0906)	-
Miscellaneous	0.388	-0.570***	-0.402***	-
	(0.320)	(0.194)	(0.0918)	-
Controls	Yes	Yes	Yes	Yes
Observations	1,352	1,517	3,376	6,071
R-squared	0.354	0.090	0.122	0.120

Robust standard errors in parentheses.

*** p<0.01

** p<0.05

* p<0.1.

The controls include dummies for the WFH frequencies after the state of the emergency, WFH frequency change, gender, age, job grades, and divisions.

The significance of the coefficients of the other variables varies across companies. We shall also note that “having responsibilities (childcare and/or nursing care)” is also negative and statistically significant for Companies A and C. During the state of emergency in April to May, a number of children did not attend school because of closures. Additionally, many daycare centers for elderly individuals have closed to avoid cluster infections of COVID-19. These closures have caused temporary loss of productivity for workers who needed to take care of their family members while working from home.

These results imply that the loss of productivity when working from home can be ameliorated by addressing those undesirable factors. In particular, the infrastructure for WFH can be relatively easily improved by appropriate IT investment or by financial support provided by companies to their employees to establish a better work environment at home. In the long run, further technological development of IT security and communication devices and learning by doing among workers will help find efficient ways to communicate within and across companies.

To address sample selection bias, we also estimated type II Tobit models (the maximum likelihood estimator and Heckman’s two-step estimator) to address potential selection into WFH as a robustness check. The estimation results did not provide evidence of selection bias and were qualitatively the same as the OLS estimation results.

### 4.3. Subsample analysis of causes

We now take a closer look at the causes of productivity losses by conducting subsample analysis. We divide the sample into four based on functional roles, i.e., corporate, sales, R&D, and production, and we estimate the model presented in Section 4.2.

Tables 5–8 present the main results. Once again, the factor that is fairly common to all four functional roles is “*poor WFH setups*,” and the coefficient estimates are significantly negative for most cases. Apparently, it may be more important for corporate and R&D jobs since the estimates are all significant, except in the case of Company A, where the estimates are significant only at the 10% level.

**Table 5:** Subsample analysis (corporate).

	Company A	Company B	Company C	Company D
	*prsnt_dif*			
Inability to retrieve data	0.211	-0.267	-0.144	-
	(0.203)	(0.198)	(0.157)	-
Inability to use exclusive equipment	-0.765***	-0.0780	0.0972	-
	(0.116)	(0.182)	(0.166)	-
Poor WFH setups	-0.686*	-0.412***	-0.378**	-0.776***
	(0.366)	(0.141)	(0.141)	(0.127)
Lack of support and/or instruction from the supervisor	0.306	-0.214	-0.147	-
	(0.411)	(0.208)	(0.219)	-
Poor workplace communication	-0.780***	-0.298	-0.314***	-0.364***
	(0.135)	(0.173)	(0.0992)	(0.133)
Poor communication with clients	-1.100***	-0.321*	-0.168	-0.493***
	(0.205)	(0.184)	(0.133)	(0.162)
Controls	Yes	Yes	Yes	Yes
Observations	402	579	522	1,621
R-squared	0.334	0.140	0.147	0.166

Robust standard errors in parentheses.

*** p<0.01

** p<0.05

* p<0.1.

The controls include the difference of WFH, dummies for the WFH frequency after the state of emergency, other perceived factors, gender, age, job grades, divisions, and functional roles.

**Table 6:** Subsample analysis (sales).

	Company A	Company B	Company C	Company D
	*prsnt_dif*			
Inability to retrieve data	-0.590***	-0.478*	0.170	-
	(0.194)	(0.247)	(0.172)	-
Inability to use exclusive equipment	-0.588***	0.00242	-0.197	-
	(0.165)	(0.525)	(0.126)	-
Poor WFH setups	-0.399*	-0.474***	-0.290	-0.394***
	(0.206)	(0.105)	(0.198)	(0.118)
Lack of support and/or instruction from the supervisor	-0.556	-0.707**	0.127	-
	(0.621)	(0.258)	(0.118)	-
Poor workplace communication	-0.180	-0.159***	-0.422**	-0.0528
	(0.244)	(0.0445)	(0.159)	(0.134)
Poor communication with clients	-1.022***	-0.385	-0.301**	-0.482***
	(0.0979)	(0.233)	(0.119)	(0.136)
Controls	Yes	Yes	Yes	Yes
Observations	444	320	468	1,536
R-squared	0.456	0.207	0.103	0.187

Robust standard errors in parentheses.

*** p<0.01

** p<0.05

* p<0.1.

The controls include the difference of WFH, dummies for the WFH frequency after the state of emergency, other perceived factors, gender, age, job grades, divisions, and functional roles.

**Table 7:** Subsample analysis (R&D).

	Company A	Company B	Company C	Company D
	*prsnt_dif*			
Inability to retrieve data	-0.925***	-0.516***	-0.108	-
	(0.153)	(0.123)	(0.0872)	-
Inability to use exclusive equipment	-0.501*	0.0137	-0.186**	-
	(0.265)	(0.205)	(0.0793)	-
Poor WFH setups	-0.645*	-0.589***	-0.524***	-0.638***
	(0.295)	(0.151)	(0.0935)	(0.186)
Lack of support and/or instruction from the supervisor	-0.575**	0.0617	-0.0519	-
	(0.235)	(0.433)	(0.126)	-
Poor workplace communication	-0.0500	0.0144	-0.353***	-0.230
	(0.292)	(0.178)	(0.0808)	(0.139)
Poor communication with clients	-1.676***	-0.541	-0.108	-0.0372
	(0.510)	(0.415)	(0.130)	(0.106)
Controls	Yes	Yes	Yes	Yes
Observations	387	342	1,427	1,186
R-squared	0.479	0.136	0.123	0.131

Robust standard errors in parentheses.

*** p<0.01

** p<0.05

* p<0.1.

The controls include the difference of WFH, dummies for the WFH frequency after the state of emergency, other perceived factors, gender, age, job grades, divisions, and functional roles.

**Table 8:** Subsample analysis (production).

	Company A	Company B	Company C	Company D
	*prsnt_dif*			
Inability to retrieve data	-0.581***	-0.294	-0.0217	-
	(0.175)	(0.235)	(0.0849)	-
Inability to use exclusive equipment	-0.464	-0.149	-0.286***	-
	(0.641)	(0.164)	(0.0998)	-
Poor WFH setups	-1.617***	-0.579*	-0.325***	-0.822**
	(0.260)	(0.305)	(0.0835)	(0.404)
Lack of support and/or instruction from the supervisor	0.279	-0.205	-0.0777	-
	(0.721)	(0.481)	(0.0820)	-
Poor workplace communication	-1.082***	0.422	-0.438***	-1.106**
	(0.288)	(0.268)	(0.0901)	(0.529)
Poor communication with clients	-0.609	-0.190	-0.0428	0.167
	(0.420)	(0.353)	(0.120)	(0.589)
Controls	Yes	Yes	Yes	Yes
Observations	115	271	959	162
R-squared	0.523	0.150	0.114	0.437

Robust standard errors in parentheses.

*** p<0.01

** p<0.05

* p<0.1.

The controls include the difference of WFH, dummies for the WFH frequency after the state of emergency, other perceived factors, gender, age, job grades, divisions, and functional roles.

Now, we turn to the specificity of each functional role. For corporate jobs and sales jobs, “*poor workplace communication*” and “*poor communication with clients*” have significantly negative effects on productivity across companies, which is consistent with the intuition that corporate jobs and sales jobs intensively involve engagement in coordination and organization both within and outside the company.

This result is reasonable considering the nature of the tasks undertaken by employees who hold these roles. For sales jobs and R&D jobs, the coefficient estimate for “*the inability to retrieve data*” is significantly negative for Companies A and B, and the coefficient estimate for “*the inability to use exclusive equipment*” is significantly negative for Companies A and C. Once again, these results are reasonable since workers engaged in R&D tend to engage with confidential information such as patents. For production jobs, the estimate for “*poor workplace communication*” is significantly negative, except in the case of Company B, and this result is also fairly consistent with the duties and tasks of workers holding such jobs.

Across functional roles, there is a common factor of productivity losses, i.e., “*poor WFH setups*,” which calls for comprehensive support for all occupations to improve the WFH conditions that employees face. In addition, our results indicate that the most important factor in improving WFH productivity differs by occupation, suggesting that employers should recognize that the optimal investment priorities may differ across occupations.

### 4.4. Frequency of WFH and mental health

We next study the relationship between mental health and WFH by estimating Eq (1). Table 9 shows the results obtained from the regression of *mental*_*health*_*i*_ on *wfh*2*d*_*i*_, *wfh*4*d*_*i*_, and *wfh*5*d*_*i*_, controlling for pre-COVID WFH experience and productivity as well as basic individual and job characteristics. The variable *wfh*_*bf*_*i*_ is a dummy indicating an individual working from home at least one day in March before the state of emergency. Prepandemic productivity, *prsnt*_*bf*_*i*_, is included to address the potential confounding factors between WFH decisions and workers’ mental health through productivity. With additional controls of divisions and job levels, we made every effort to account for potential confounding factors because the outcome variable is not the first difference, unlike the estimates for presenteeism; thus, unobservable worker characteristics could still bias the results.

**Table 9:** Regression of mental health on WFH frequency.

	Company B	Company C	Company D
	*mental_health*		
*wfh_5d*	0.182**	0.189**	0.109***
	(0.0801)	(0.0736)	(0.0360)
*wfh_4d*	0.107	0.138**	0.177***
	(0.0633)	(0.0600)	(0.0324)
*wfh_2d*	0.0678	0.0736	0.0770***
	(0.0415)	(0.0571)	(0.0279)
*wfh2_bf*	0.0228	-0.0280	-0.0997***
	(0.0490)	(0.0441)	(0.0275)
*female w/o children*	0.00197	0.118*	-0.189***
	(0.0707)	(0.0649)	(0.0289)
*female w/ children*	-0.0250	0.238**	-0.220***
	(0.141)	(0.0991)	(0.0470)
*age30*	-0.198***	0.148**	0.0853**
	(0.0670)	(0.0632)	(0.0371)
*age40*	-0.0722	0.0893	0.176***
	(0.0791)	(0.0549)	(0.0390)
*age50*	0.0341	0.238***	0.233***
	(0.0566)	(0.0646)	(0.0378)
*age60*	0.455***	0.419***	0.541***
	(0.0503)	(0.0819)	(0.0537)
*prsnt_dif*	0.0409***	0.102***	0.0241***
	(0.00887)	(0.0149)	(0.00418)
Constant	0.110	0.0566	-0.521***
	(0.0821)	(0.0981)	(0.0633)
Controls	Yes	Yes	Yes
Observations	2,755	3,720	10,636
R-squared	0.071	0.088	0.069

Robust standard errors in parentheses.

*** p<0.01

** p<0.05

* p<0.1.

The controls include job grades, functional roles, and sections.

Overall, employees’ mental health seems to have a positive association with the frequency of WFH, implying that WFH could mitigate the mental deterioration of workers. The relationship is particularly significant at Company D, where the sample size is largest. However, the overall pattern is similar among the three firms.

The greatest concern for the result is sample selection, particularly in Companies B and D, where the response rate is lowest. It may be the case that employees whose mental health is damaged by WFH were less likely to respond to the survey request than those who benefit from WFH or those whose productivity is damaged by not WFH. Such selection could impose upward bias on the coefficient of WFH variables. Although we do not rule out this possibility, we observe a surprisingly similar pattern among the three firms whose labor-management relationships should vary. This implies that selection bias might be relatively small.

### 4.5. Costs and Benefits of WFH

To identify what factors contribute to improvements and deteriorations in mental health, we estimate Eq (1), adding as explanatory variables the responses to the question of what factors the respondents perceived as advantages and disadvantages of WFH and restricting the sample to those who worked from home after the state of emergency. We shall note that we estimated a sample selection model for mental health and WHF, but the evidence of selection bias was weak, and the estimates remained substantially identical.

The factors that have a strong association with better mental health, conditional on individual and job characteristics, should be the main benefits of WFH. Two potential benefits emerge from the results shown in Table 10. First, the coefficients of “*better sleep*” and “*drinking less alcohol*” are significantly positive across companies. Second, “*can avoid unnecessary communication*”, “*facilitates a greater focus on work*”, and “*enjoying stay　home*” are significantly associated with better mental health for Companies C and D, although a similar pattern cannot be observed for Company B. Notably, “*zero commuting and saving time*” is significantly positive for Company D.

**Table 10:** Regression of mental health on the perceived advantages and disadvantages of WFH.

	Company B	Company C	Company D
	* *	*mental_health*	* *
Better sleep	0.136**	0.145***	0.166***
	(0.0615)	(0.0313)	(0.0217)
Drinking less alcohol	0.247***	0.194***	0.0792*
	(0.0455)	(0.0512)	(0.0475)
Can avoid unnecessary communication	-0.00981	0.0535**	0.0820***
	(0.0325)	(0.0252)	(0.0131)
Faciliates a greater focus on work	0.0356	0.122***	0.107***
	(0.0354)	(0.0345)	(0.0353)
Enjoying staying home	0.0138	0.0955***	0.127***
	(0.0434)	(0.0292)	(0.0203)
Zero commuting and saving time	0.0630	0.0509	0.100***
	(0.0392)	(0.0448)	(0.0309)
Exercising more	0.0635	0.0310	0.0740***
	(0.0433)	(0.0249)	(0.0182)
Improvement in IT skills	-0.0939	0.0415	0.0808***
	(0.0689)	(0.0405)	(0.0232)
Project delay	-0.257***	-0.325***	-0.179***
	(0.0584)	(0.0396)	(0.0226)
Poor IT environment	-0.162**	-0.110***	-0.0646**
	(0.0640)	(0.0310)	(0.0294)
Musculoskeletal pain	-0.137***	-0.129***	-0.163***
	(0.0275)	(0.0389)	(0.0314)
Eye strain	-0.173***	-0.123***	-0.107***
	(0.0474)	(0.0394)	(0.0353)
Migraine	-0.398**	-0.623***	-0.373***
	(0.171)	(0.0790)	(0.0643)
Having to prepare meals	-0.108**	-0.0183	-0.151***
	(0.0445)	(0.0449)	(0.0332)
Weight gain	-0.0815**	-0.0529	-0926***
	(0.0378)	(0.0332)	(0.0341)
Childcare due to school closure	-0.164**	-0.0801	-0.0396
	(0.0793)	(0.0631)	(0.0336)
Nursing care for parents	0.0608	-0.371**	-0.215*
	(0.378)	(0.162)	(0.118)
Controls	Yes	Yes	Yes
Observations	1,498	3,409	4,026
R-squared	0.199	0.268	0.306

Robust standard errors in parentheses.

*** p<0.01

** p<0.05

* p<0.1.

The controls include dummies for the WFH frequency after the state of emergency, a dummy for WFH experience in March, the pre-pandemic productivity, other perceived advantages and disadvantages, gender, age, job grades, and divisions.

The result suggests that WFH improves the quality of sleep presumably by reducing time to prepare for work and to commute. Interestingly, it also reveals that less drinking is positively associated with mental health. This may reflect the fact that workers find WFH benefits since they do not have to go for a drink with colleagues after work or because improved mental health due to WFH induces the workers to consume less alcohol. Additionally, due to fewer interruptions that would normally occur at the workplace, WFH allows for a quieter environment that can facilitate a greater focus on work. Although undesirable aspects of WFH are oftentimes emphasized by business practitioners, WFH may improve productivity by improving employees’ health and well-being.

This benefit due to the longer rest period enabled by WFH should have the same impact as shorter working hours. In fact, in the literature, there is some evidence of the benefits of shorter working hours. Using data on women working in manufacturing plants to produce artillery shells for the British military during the First World War, Pencavel [37] found that the hours-productivity profile exhibits a concave, nonmonotonic shape, implying that having a longer rest period could improve productivity when workers work excessive hours. Similarly, using single-company data on Japanese construction design projects, Shangguan et al. [38] showed that team productivity and the quality of work improved when working hours were reduced during the great recession.

In contrast, there seem to be negative factors for employees working from home that worsen their mental health. The coefficients for “*project delay*” and “*poor IT environment*” are significantly negative across companies. Remarkably, physical disorders such as “*musculoskeletal pain*”, “*eye strain*”, and “*migraine*” are significantly associated with deteriorated mental health. These findings are consistent with Moretti et al. [29] who claim that WFH causes musculoskeletal pain that reduces productivity (see also [30, 31]).

These estimates suggest that employees are frustrated with delays in ongoing projects, possibly due to poor IT setups. As a result, frustration and poor IT setups might stress employees and damage both their physical health and mental health. This result presents the need to support the establishment of physically friendly WFH facilities and IT infrastructures.

### 4.6. Gender differences

There are some studies reporting a worse labor market outcome (Adams-Prassl et al. [20]) and larger declines in productivity among women than men (Etheridge et al. [19]) or with worse mental health (Felstead and Reuschke [6], Etheridge and Spantig [39]) for female workers during the pandemic. Most attribute the difference to women’s increased role in family and caring responsibilities (see also [40]). To this end, we paid special attention to female dummies in all of our analyses and conducted subsample analyses to examine whether women are affected by WFH during the pandemic in any different way than men. Surprisingly, we did not find any consistent gender differences among the four firms. For example, while the productivity of female employees was significantly higher than that of their male counterparts (Table 4), their mental health was significantly worse in Company D (Table 9). In contrast, the mental health of female employees was significantly better than that of their male counterparts in Company C, and we did not find any statistically significant gender differences in Companies A and B (Tables 4 and 9).

Expecting that there might be heterogeneity among female employees depending on the family structure (e.g., the number and ages of children), we conducted subsample analysis of Tables 4 and 10 with or without additional controls of the number and ages of children to see the effects of WFH by gender. However, we did not obtain any consistent differences between men and women among the four firms regarding the number and ages of children, or the costs and benefits of WFH on productivity and mental health, although there were some idiosyncratic gender differences that were specific to one firm.

One obvious reason for the difference reported in other studies and ours is that, in a representative sample, female workers are more likely to be unemployed or face wage cuts during the pandemic since many of them are working in the service industry. In contrast, workers in our analysis are from large industrial companies with relatively higher education and are more likely to be engaged in technical or professional jobs; therefore, they have less fear of job security or wages. We also speculate that women can do more of their tasks from home, especially those with school-age children, because they are more likely to be assigned routine or more narrowly defined standardized tasks with less coordination needs, which is different from Adams-Prassl et al. [21] who find that women can do fewer tasks from home even within occupations and industries in the US and UK. Using personnel records from a large Japanese manufacturing company, Sato et al. [40] show that there is a substantial gender difference in developmental job assignment, which presumably comes from different time preferences/constraints between men and women. If female employees can work from home more easily, they will not necessarily be more negatively affected by WFH despite their additional burden in family care during the pandemic.

## 5. Concluding remarks

Using unique data retrieved from our original survey conducted in cooperation with four manufacturing companies in Japan, we investigated the determinants of the quality of WFH under the COVID-19 pandemic. Specifically, we examined the within-company and within-occupation productivity effects of WFH on employees’ productivity and mental health. Focusing on specific companies also allowed us to exclude the differences in productivity among firms.

We present four findings. First, we confirmed that frequent WFH is associated with decreased productivity. In our interpretation, most workers experienced declines in productivity, probably due to their inadequate preparation for WFH under the sudden shock of the pandemic.

Second, to confirm our interpretation, we identified the possible factors of productivity losses during pandemic-driven WFH. Our estimation results suggest that the major contributors to deteriorations in productivity are poor WFH setups and poor communication at the workplace and with clients. These results imply that companies may enhance employees’ productivity by investing in their WFH setups at home and communication tools.

Third, we also examined the heterogeneity across types of jobs. We categorized occupational categories into four functional roles, i.e., corporate, sales, R&D, and production. We have found that poor WFH setups are one of the major causes of productivity losses across the four occupation types. However, there are also several important causes that are specific to certain occupations. For corporate jobs and sales jobs, poor workplace communication and poor communication with clients seem to be the most crucial. For sales and R&D jobs, the lack of access to crucial information and exclusive equipment appear to contribute to productivity losses. Our findings provide managerial implications that are useful for designing desirable investments to improve employees’ productivity while WFH.

Fourth, our results show that WFH is associated with better employee mental health. Our regression results suggest that workers benefit from a greater focus on work with a quieter environment, less fatigue, and additional time for sleep and rest as a result of the time saved by cutting commuting time. The positive association between WFH and mental health, which is not in line with some early works on the effect of COVID-19 on mental health, may come from two factors: (1) the movement and social life were less restricted in Japan during the pandemic; and (2) WFH was not mandated so that only organizations that can allow and provide support for WFH actually implemented it. Since a lack of time series information on mental health prevents us from ruling out a time-invariant component of employees’ mental status, however, the findings here should be handled with reservations. Nonetheless, while more emphasis tends to be placed on the drawbacks of WFH, our result may suggest that WFH may improve productivity by improving employees’ health and well-being. To that end, let us introduce the answers to the question regarding WFH used in the Company A surveys. The question asked, “*(a)fter the situation returns to normal*, *how often do you prefer to work from home*?*”* Among 1,381 employees who worked from home, only 7.2% answered “none,” while 52.3% and 22.0% answered “1–2 days per week” and “3 days or more per week”, respectively. These results suggest that these workers might have realized the advantages of WFH, and they are in line with the results of Eurofound’s questionnaire survey [2] conducted with workers in EU member states. The WFH style may take root around the world as a new working style.

Under these circumstances, companies should not dismiss remote working out of hand as a work arrangement option because of lower productivity compared with in-office work. Rather, they need to conduct a detailed analysis of the causes of the productivity gap, make the infrastructure improvements that are necessary for increasing WFH productivity, and send a clear message from top management that WFH can be a productivity booster. Such changes will create opportunities for people who have been unable to work full-time or work as regular employees—that is, employees who are supposed to be willing to make business trips or accept workplace transfers—because of time constraints resulting from life circumstances, such as having to raise children or care for elderly individuals or individuals suffering from illness or a disability. In a way, WFH may be an option that can be used to take full advantage of the workforce’s talents that could be wasted without such arrangement.

## Supporting information

S1 Table Descriptive statistics. (DOCX)
