# Supplementary material for: Working from home and productivity under the COVID-19 pandemic: Using survey data of four manufacturing firms
Source: PLoS One. 2021 Dec 23;16(12):e0261761. doi: 10.1371/journal.pone.0261761 (PMC8700052; doi:10.1371/journal.pone.0261761)
Supplement: S1 Table — (DOCX) [file pone.0261761.s001.docx]

**Table S1. Descriptive statistics**
